# Supplementary material for: Snakebite patterns in rural Sri Lanka and their implications for preventive measures
Source: PLoS Negl Trop Dis. 2026 Mar 9;20(3):e0014092. doi: 10.1371/journal.pntd.0014092 (PMC12991362; doi:10.1371/journal.pntd.0014092)
Supplement: S1 Table — (PDF) [file pntd.0014092.s001.pdf]

S1 Table: Bite patterns of unidentified snakes

	**Unidentified snakebites**
**Age**	
Median (years)	42
IQR	27-54
**Gender**	**(N=2480)**
Male	1619 (65·3%)
Female	861 (34·7%)
**Location of bite**	**(N=2295)**
Domestic gardens	793 (34·5%)
Indoors	423 (18·3%)
Farmlands	614 (26·5%)
Roadside	222 (9·7%)
Jungle	20 (0·9%)
Other locations	223 (9·7%)
**Anatomical site of the bite**	**(N=2441)**
Foot	1636 (67·0%)
Hand	388 (15·9%)
Ankle	88 (3·6%)
Leg	205 (8·4%)
Other sites	124 (5·1%)
**Activity while the bite occurred**	**(N=2350)**
Walking	1260 (53·6%)
Agricultural work	192 (8·2%)
Cleaning	123 (1·9%)
Sleeping	201 (8·5%)
Collecting firewood	28 (1·2%)
Other activity	546 (23·2%)
**Diurnal variation**	**(N=2463)**
06:00 to 18:00	1073 (43·6%)
18:00 to 06:00	1390 (56·4%)
**Provocation of the snake by touching**	**(N=2495)**
	164 (6·6%)
