# Supplementary material for: Snakebite patterns in rural Sri Lanka and their implications for preventive measures
Source: PLoS Negl Trop Dis. 2026 Mar 9;20(3):e0014092. doi: 10.1371/journal.pntd.0014092 (PMC12991362; doi:10.1371/journal.pntd.0014092)
Supplement: S2 Table — (PDF) [file pntd.0014092.s002.pdf]

S2 Table: Snakebite patterns in common bite locations with relation to the rainy season and non-rainy season

	Domestic gardens		Farmlands		Indoors	
	**Rainy season (October to February)**	**Non-rainy season (March to September)**	**Rainy season (October to February)**	**Non-rainy season (March to September)**	**Rainy season (October to February)**	**Non-rainy season (March to September)**
**Gender**	**(N=806)**	**(N=846)**	**(N=630)**	**(N=523)**	**(N=408)**	**(N=461)**
Male	438 (54·3%)	467 (55·2%)	542 (86·0%)	440 (84·0%)	188 (46·1%)	205 (44·5%)
Female	368 (45·7%)	379 (44·8%)	88 (14·0%)	83 (16·0%)	220 (53·9%)	256 (55·5%)
**Activity while the bite occurred**	**(N=776)**	**(N=861)**	**(N=615)**	**(N=518)**	**(N=407)**	**(N=459)**
	Walking 444 (55·6%)	Walking 486 (57·7%)	Farming 287 (46·7%)	Farming 237 (45·8%)	Walking 158 (38·8%)	Walking 183 (39·9%)
	Gardening 156 (19·5%)	Gardening 160 (19·0%)	*Harvesting* 97 (15·7%)	*Harvesting* 91 (17·5%)	Sleeping 82 (20·2%)	Sleeping 141 (30·7%)
	Collecting firewood 45 (5·6%)	Collecting firewood 37 (4·4%)	*Ground preparation* 41 (6·6%)	*Ground preparation* 15 (2·9%)	Cleaning 24 (5·9%)	Cleaning 16 (3·5%)
	Sleeping 2 (0·3%)	Sleeping 16 (1·9%)	*Irrigation* 15 (2·4%)	*Irrigation* 20 (3·8%)	Handling firewood 24 (5·9%)	Handling firewood57 (1·1%)
	Bathing/ washing ··	Bathing/ washing 6 (0·7%)	*Other agricultural work* 134 (21·7%)	*Other agricultural work* 111 (21·4%)	Bathing 6 (1·5%)	Bathing 2 (0·4%)
	Other 152 (19·0%)	Other 137 (16·3%)	Walking 277 (45·0%)	Walking 240 (46·3%)	Other 123 (30·2%)	Other 112 (24·4%)
			Sleeping 29 (4·7%)	Sleeping 20 (3·9%)		
			Other 22 (3·6%)	Other 21 (4·1%)		
**Anatomical site of the bite**	**(N=780)**	**(N=860)**	**(N=620)**	**(N=519)**	**(N=401)**	**(N=453)**
Foot	528 (66·0%)	554 (65·6%)	447 (72·1%)	387 (74·6%)	205 (51·1%)	254 (56·1%)
Hand	205 (25·6%)	172 (20·4%)	69 (11·1%)	38 (7·3%)	133 (33·2%)	115 (25·4%)
Leg	28 (3·5%)	62 (7·4%)	42 (6·8%)	47 (9·1%)	19 (4·7%)	19 (4·2%)
Ankle	23 (2·9%)	45 (5·3%)	32 (5·2%)	26 (5·0%)	16 (4·0%)	9 (2·0%)
Other	16 (2·0%)	11 (1·3%)	30 (4·8%)	21 (4·0%)	28 (7·0%)	56 (12·3%)
**Authenticated snake species**	**(N=434)**	**(N=424)**	**(N=285)**	**(N=254)**	**(N=220)**	**(N=226)**
Hypnale hypnale	276 (66·0%)	248 (56·1%)	76 (26·7%)	55 (21·7%)	104 (47·3%)	66 (29·2%)
Daboia russelii	78 (18·7%)	126 (28·5%)	176 (61·7%)	181 (71·3%)	9 (4·1%)	32 (14·2%)
Bungarus caeruleus	9 (2·2%)	9 (2·0%)	6 (2·1%)	8 (3·1%)	17 (7·7%)	42 (18·6%)
Naja naja	10 (2·4%)	11 (2·5%)	13 (4·6%)	2 (0·8%)	4 (1·8%)	13 (5·7%)
Mild and non-venomous	45 (10·7%)	48 (10·9%)	14 (4·9%)	8 (3·1%)	86 (39·1%)	73 (32·3%)
**Peak time period**	3 pm to 6 pm (258, 31·9%)	6 pm to 9 pm (286, 33·8%)	9 am to 12 pm (146, 23·3%)	6 pm to 9 pm (127, 24·4%)	3 pm to 6 pm (106, 26·2%)	6 pm to 9 pm (121, 26·3%)
