# Supplementary material for: Snakebite patterns in rural Sri Lanka and their implications for preventive measures
Source: PLoS Negl Trop Dis. 2026 Mar 9;20(3):e0014092. doi: 10.1371/journal.pntd.0014092 (PMC12991362; doi:10.1371/journal.pntd.0014092)
Supplement: S3 Table — (PDF) [file pntd.0014092.s003.pdf]

S3 Table: Snakebite patterns in common bite locations with relation to the daytime and nighttime.

	Domestic gardens		Farmlands		Indoors	
	**Day 06:00 to 18:00**	**Night 18:00 to 06:00**	**Day 06:00 to 18:00**	**Night 18:00 to 06:00**	**Day 06:00 to 18:00**	**Night 18:00 to 06:00**
**Gender**	**(N=781)**	**(N=867)**	**(N=727)**	**(N=421)**	**(N=267)**	**(N=597)**
Male	402 (51·5%)	500 (57·7%)	587 (80·7%)	389 (92·4%)	96 (36·0%)	295 (49·4%)
Female	379 (48·5%)	367 (42·3%)	140 (19·3%)	32 (7·6%)	171 (64·0%)	302 (50·6%)
**Activity while the bite occurred**	**(N=776)**	**(N=861)**	**(N=715)**	**(N=412)**	**(N=265)**	**(N=596)**
	Walking 294 (37·9%)	Walking 632 (73·4%)	Farming 378 (52·9%)	Farming 144 (35·0%)	Walking 33 (27·6%)	Walking 268 (45·0%)
	Gardening 262 (33·8%)	Gardening 54 (6·3%)	*Harvesting* 138 (19·3%)	*Harvesting* 50 (12·1%)	Sleeping 36 (13·6%)	Sleeping 182 (30·5%)
	Collecting firewood 52 (6·7%)	Collecting firewood 30 (3·5%)	*Ground preparation* 48 (6·7%)	*Ground preparation* 7 (1·7%)	Cleaning 28 (10·6%)	Cleaning 12 (2·0%)
	Sleeping 4 (0·5%)	Sleeping 14 (1·6%)	*Irrigation* 18 (2·5%)	*Irrigation* 17 (4·1%)	Handling firewood 12 (4·5%)	Handling firewood 7 (1·2%)
	Bathing/ washing 4 (0·5%)	Bathing/ washing 5 (0·6%)	*Other agricultural work* 174 (24·3%)	*Other agricultural work* 70 (17·0%)	Bathing 3 (1·1%)	Bathing 5 (0·8%)
	Other 160 (20·6%)	Other 126 (14·6%)	Walking 304 (42·5%)	Walking 210 (51·0%)	Other 113 (42·6%)	Other 122 (22·5%)
			Sleeping 7 (1·0%)	Sleeping 41 (9·9%)		
			Other 26 (3·6%)	Other 17 (4·1%)		
**Anatomical site of the bite**	**(N=780)**	**(N=860)**	**(N=716)**	**(N=417)**	**(N=262)**	**(N=587)**
Foot	443 (56·8%)	635 (73·8%)	526 (73·5%)	304 (72·9%)	105 (40·1%)	353 (60·1%)
Hand	265 (34·0%)	112 (13·0%)	73 (10·2%)	33 (7·9%)	120 (45·8%)	125 (21·3%)
Leg	33 (4·2%)	57 (6·6%)	59 (8·2%)	30 (7·2%)	10 (3·8%)	28 (4·8%)
Ankle	27 (3·5%)	41 (4·8%)	35 (4·9%)	23 (5·5%)	10 (3·8%)	15 (2·6%)
Other	12 (1·5%)	15 (1·8%)	23 (3·2%)	27 (6·5%)	17 (6·5%)	66 (11·2%)
**Authenticated snake species**	**(N=434)**	**(N=424)**	**(N=360)**	**(N=179)**	**(N=153)**	**(N=293)**
Hypnale hypnale	272 (62·7%)	250 (58·9%)	78 (21·7%)	53 (29·6%)	68 (44·4%)	102 (34·8%)
Daboia russelii	113 (26·0%)	91 (21·5%)	251 (69·7%)	106 (59·3%)	5 (3·3%)	36 (12·3%)
Bungarus caeruleus	5 (1·1%)	13 (3·1%)	3 (0·8%)	11 (6·1%)	12 (7·8%)	47 (16·0%)
Naja naja	15 (3·5%)	6 (1·4%)	14 (3·9%)	1 (0·6%)	13 (8·5%)	4 (1·4%)
Mild and non-venomous	29 (6·7%)	64 (15·1%)	14 (3·9%)	8 (4·4%)	55 (36·0%)	104 (35·5%)
**Peak time period**	3pm-6pm (246, 31·4%)	6pm-9pm (544, 62·5%)	3pm-6pm (249, 34·2%)	6pm-9pm (254, 60·3%)	3pm to 6pm (77, 28·8%)	6pm to 9pm (227, 38·0%)
