# Supplementary material for: Snakebite patterns in rural Sri Lanka and their implications for preventive measures
Source: PLoS Negl Trop Dis. 2026 Mar 9;20(3):e0014092. doi: 10.1371/journal.pntd.0014092 (PMC12991362; doi:10.1371/journal.pntd.0014092)
Supplement: S4 Table — (PDF) [file pntd.0014092.s004.pdf]

S4 Table: Analysis based on the highest education level

	Up to Grade 10 (n=3699)	Above Grade 10 (n=766)
**Age**	**(n=3681)**	**(n=762)**
Median (years)	44	33
IQR	30-55	25-46
**Gender**	**(n=3690)**	**(n=764)**
Male	2430 (65·9%)	444 (58·1%)
Female	1260 (34·1%)	320 (41·9%)
**Location of bite**	**(n=2295)**	**(n=731)**
Domestic gardens	1316 (36·6%)	316 (43·2%)
Indoors	665 (18·5%)	186 (25·4%)
Farmlands	1009 (27·3%)	112 (15·3%)
Roadside	296 (8·2%)	57 (7·8%)
Other locations	307 (8·5%)	60 (8·2%)
**Anatomical site of the bite**	**(n=3647)**	**(n=756)**
Foot	2593 (71·1%)	531 (70·2%)
Hand	683 (18·7%)	147 (19·4%)
Leg	224 (6·1%)	50 (6·6%)
Other sites	147 (4·1%)	28 (3·7%)
**Activity while the bite occurred**	**(n=3656)**	**(n=755)**
Walking	1862 (50·9%)	435 (57·6%)
Agricultural work	409 (11·2%)	38 (5·0%)
Cleaning	294 (8·0%)	79 (10·5%)
Sleeping	250 (6·8%)	31 (4·1%)
Collecting firewood	89 (2·4%)	21 (2·8%)
Other activity	752 (20·6%)	151 (20·0%)
