# Supplementary material for: Snakebite patterns in rural Sri Lanka and their implications for preventive measures
Source: PLoS Negl Trop Dis. 2026 Mar 9;20(3):e0014092. doi: 10.1371/journal.pntd.0014092 (PMC12991362; doi:10.1371/journal.pntd.0014092)
Supplement: S5 Table — (PDF) [file pntd.0014092.s005.pdf]

S5 Table: Anatomical site of snakebites in farmlands with type of agricultural activity

	Agricultural activity				
**Bite site**	**Harvesting**	**Ground preparation**	**Irrigation**	**Unspecified**	**Total**
Foot	144 (77·8%)	44 (78·6%)	29 (82·9%)	160 (65·8%)	377 (72·6%)
Hand	18 (9·7%)	5 (8·9%)	4 (11·3%)	44 (18·1%)	71 (13·7%)
Leg	10 (5·4%)	2 (3·6%)	1 (2·9%)	19 (7·8%)	32 (6·2%)
Ankle	4 (2·2%)	4 (7·1%)	1 (2·9%)	11 (4·5%)	20 (3·9%)
Other	9 (4·9%)	1 (1·8%)	··	9 (3·7%)	19 (3·7%)
