# Supplementary material for: Snakebite patterns in rural Sri Lanka and their implications for preventive measures
Source: PLoS Negl Trop Dis. 2026 Mar 9;20(3):e0014092. doi: 10.1371/journal.pntd.0014092 (PMC12991362; doi:10.1371/journal.pntd.0014092)
Supplement: S6 Table — (PDF) [file pntd.0014092.s006.pdf]

S6 Table: Studies describing snakebite proportion of snake species in dry zone, Sri Lanka

**Study**	**De Silva, Anslem (1981), Snake-bite in Anuradhapura district**	**Data from MOH, Kekirawa (obtained from De Silva, A. and Ranasinghe, L. (1983), Epidemiology of snake-bite in Sri Lanka; a Review)**	**Kasturiratne, A et al (2005), Estimates of disease burden due to land-snake bite in Sri Lankan hospitals**	**Whitehall JS et al (2007), Snake bites in north east Sri Lanka**	**Pirasad S. et al (2015), Epidemic Poisoning with Snakes in Eastern Sri Lanka; Epidemiologic al and Clinical Features in Batticaloa District**	**Jayawardana S. et al (2020), Prevalence, vulnerability and epidemiological characteristics of snakebite in agricultural settings in rural Sri Lanka: A population-based study from South Asia**
Study period	January to December 1979	August 1981 to August 1982	2000	January to November 2005	-	-
Geographical region	Anuradhapura	MOH area, Kekirawa, Anuradhapura	Dry zone (including Anuradhapura)	Dry zone (Kilinochchi, Sri Lanka)	Dry zone (Batticaloa, Sri Lanka)	Dry zone (Ampara, Sri Lanka)
Merrem's hump-nosed pit viper	84 (22·2%)	22 (16·9%)	1-10%	-	-	23 (21·5%)
Russell's viper	115 (30·3%)	64 (49·2%)	45-55%	14 (14%)	25 (24%)	16 (14·9%)
Indian krait	56 (14·8%)	17 (13·1%)	25-35%	6 (6%)	42 (40·1%)	4 (3·7%)
Indian cobra	65 (17·1%)	27 (20·8%)	1-10%	6 (6%)	18 (17·3%)	-
Non and mildly venomous	58 (15·3%)	-	5-15%	-	-	64 (59·8%)
