# Supplementary material for: Snakebite patterns in rural Sri Lanka and their implications for preventive measures
Source: PLoS Negl Trop Dis. 2026 Mar 9;20(3):e0014092. doi: 10.1371/journal.pntd.0014092 (PMC12991362; doi:10.1371/journal.pntd.0014092)
Supplement: S7 Table — (DOCX) [file pntd.0014092.s007.docx]

**S7 Table: Findings of studies describing snakebite patterns of venomous snake species in Anuradhapura. ‘*’ – Studies describe the snakebite patterns collectively from different geographical regions of the country.**

|  | **Hump-nosed viper** | | **Russell’s viper** | | **Indian krait** | | **Indian Cobra** |
| --- | --- | --- | --- | --- | --- | --- | --- |
| Study | Ariyaratnam CA et al (2008)[1], Frequent and potentially fatal envenoming by hump-nosed pit vipers (*Hypnale hypnale* and *H.nepa*) in Sri Lanka: lack of effective antivenom* | Maduwage K et al (2013)[2], Epidemiology and clinical effects of hump-nosed pit viper  (Genus: *Hypnale*) envenoming in Sri Lanka* | Kularatne SAM (2003)[3], Epidemiology and the clinical picture of the Russell’s viper (*Daboia russelii russelii*) bite in Anuradhapura, Sri Lanka; A prospective study of 336 patients | Kularatne et al (2014)[4], Revisiting Russell’s viper (*Daboia russelli*) bite in Sri Lanka; Is abdominal pain an early feature of systemic envenoming? | Kularatne SAM (2002)[5], Common krait (*Bangarus caeruleus*) bite in Anuradhapura, Sri Lanka; a prospective clinical study, 1996-98 | Ariaratnam CA (2008)[6], Distinctive Epidemiologic and Clinical Features of Common Krait (Bungarus caeruleus)  Bites in Sri Lanka* | Kularatne SAM (2009)[7], Epidemiology, clinical profile and management issues of cobra (*Naja naja*) bites in Sri Lanka: first authenticated case series* |
| Study period | August 1993 to July 1997 | July 2008 to July 2010 | January 1996 to December 1997 | January to December 2010 | January 1996 to December 1998 | August 1993 to July 1997 | Anuradhapura (dry zone) from 1995 to  1998 Peradeniya (wet zone) from 2002 to  2007 |
| Number of patients | 302 (*H. hypnale* bites 301, *H. nepa* bite 1) (62 patients from Anuradhapura) | 114 ( *H. hypnale* 93 (81%), *H. zara* 16 (14%), *H. nepa* 5 (4%) from 6 hospitals including Teaching Hospital Anuradhapura) | 336 | 55 (specimen-authenticated) | 210 | 88 (from 7 hospitals including Teaching Hospital, Anuradhapura) | 25 specimen authenticated (19 from Anuradhapura, 6 from Peradeniya) |
| Gender |  |  |  |  |  |  |  |
| Male | 49% | 75 (66%) | 267 (80%) | 81% | 50% | 61% | 10 (40%) |
| Female | 51% | 39 (34%) | 69 (20%) | 19% | 50% | 39% | 15 (60%) |
| Age (Years) | 21-30, 78 (25.8%) | Median 39 | 10-40, 256 (77%) | 20-50 (72.7%) | 10-30 (52%) | 11-40, 67 (76%) | Median – 36 (Anuradhapura) |
| Bite location | Patients compound 148 (48%)  Road/footpaths 90 (30%)  Paddy field 33 (11%)  Indoors 6 (2%) | “Most of *H. hypnale*  bites were in home gardens” | Paddy fields 136 (41%)  Footpath 98 (29%)  Home garden 64 (19%)  Indoor 3(0.8%) | Chena 53%  Paddy fields 5.5%  Residential compound 20%  Roadside 20% | “at night while the victims were sleeping on the floor” | Indoors 87 (98.9%) | Compound 13 (52%)  Footpath 3 (12%)  Paddy field 3 (12%)  Forest 1 (4%) |
| The site of the bite | Lower limbs 246 (81%)  Upper limb 56 (19%) | Lower limb 78 (68%) | Foot 281 (84%)  Ankle-leg 41 (9%)  Hand-arm 10 (%) | - | Hand 50 (30%)  Foot 27 (16%)  Upper arm 20 (12%)  Neck 5 (3%) | Upper  limbs 31(35%)  lower limbs  22 (25%)  Buttocks 17 (19%)  head and neck 7 (8%) | Lower limb 15 (60%)  Upper limb 10 (40%) |
| Activity while the bite occurred | Walking on roads/footpaths 142 (47%)  Gardening,47 (16%)  Farming, 33 (11%)  Other work, 33  Collecting firewood, 17 (6%)  Sleeping 4(1%) | - | - | - | “at night while the victims were sleeping on the floor” | “all were asleep inside mud or clay  houses thatched with woven dried coconut palm leaves (cad  jan) in rural areas except one” | - |
| -Diurnal variation | 00:00—06:00, 17 (6%)  06:00—12:00, 48 (16%)  12:00—18:00, 86 (28%)  18:00—00:00h, 151 (50%) | “Most bites occurred in the daytime” | 08:00 – 12:00 70 (21%)  18:00 – 22:00 133 (40%) | 12:00 – 21:00 (67%) | 22:00 – 04:00 (66.6%) | 23:00–24:00, 22 (25%)  24:00–01:00, 19 (22%)  01:00 -  02:00, 17 (19%)  02:00–03:00, 18 (20%)  03:00–14:00, 8(9%)  04:00–05:00, 4(5%) | 06:00 – 18:00 17 (89%) (Anuradhapura) |
| Seasonal variation | “Bites occurred throughout the year” | - | Two peaks  March-April (28%)  October- November (19%) | Two peaks  March  November | Rainy season 103 (49%) (Most number September to December) | September–October 59 (67%) | - |

References

1. Ariaratnam CA, Thuraisingam V, Kularatne SAM, Sheriff MHR, Theakston RDG, de Silva A, et al. Frequent and potentially fatal envenoming by hump-nosed pit vipers (*Hypnale hypnale* and *H. nepa*) in Sri Lanka: lack of effective antivenom. *Trans R Soc Trop Med Hyg*. 2008; **102(11)**:1120–6.
2. Maduwage K, Isbister GK, Silva A, Bowatta S, Mendis S, Gawarammana I. Epidemiology and clinical effects of hump-nosed pit viper (Genus: *Hypnale*) envenoming in Sri Lanka. *Toxicon Off J Int Soc Toxinology*. 2013; **61**:11–5.
3. Kularatne SAM. Epidemiology and clinical picture of the Russell’s viper (Daboia russelii russelii) bite in Anuradhapura, Sri Lanka: a prospective study of 336 patients. *Southeast Asian J Trop Med Public Health* 2003; **34(4)**: 855–62.
4. Kularatne SAM, Silva A, Weerakoon K, Maduwage K, Walathara C, Paranagama R, et al. Revisiting Russell’s viper (*Daboia russelii*) bite in Sri Lanka: is abdominal pain an early feature of systemic envenoming? *PloS One*. 2014; **9(2)**:e90198.
5. Kularatne SAM. Common krait (Bungarus caeruleus) bite in Anuradhapura, Sri Lanka: a prospective clinical study, 1996-98. *Postgrad Med J* 2002; **78(919)**: 276–80.
6. Ariaratnam CA, Sheriff MHR, Theakston RDG, Warrell DA. Distinctive epidemiologic and clinical features of common krait (*Bungarus caeruleus*) bites in Sri Lanka. *Am J Trop Med Hyg*. 2008; **79(3)**: 458–62.
7. Kularatne SAM, Budagoda BDSS, Gawarammana IB, Kularatne WKS. Epidemiology, clinical profile and management issues of cobra (*Naja* *naja*) bites in Sri Lanka: first authenticated case series. *Trans R Soc Trop Med Hyg.* 2009; **103(9)**: 924–30.
