# Supplementary material for: Snakebite patterns in rural Sri Lanka and their implications for preventive measures
Source: PLoS Negl Trop Dis. 2026 Mar 9;20(3):e0014092. doi: 10.1371/journal.pntd.0014092 (PMC12991362; doi:10.1371/journal.pntd.0014092)
Supplement: Attachment — Submitted filename: Response to the reviewers.docx [file pntd.0014092.s010.docx]

**Response to Reviewers**.

**Reviewer #1:**

**Methods**
No new analyses required

**Results**
Yes (No comments)

**Conclusion**
Yes - but i would like more information on obtaining impact. otherwise risks just being a paper that describes and does not change anything

Response:

We have included additional information regarding on the impact in the relevant section of the manuscript.

**Summary and general comments**

This manuscript reports 4708 snake bites over around 7 years, of which 2202 were species authenticated and analysed more carefully. The authors identify species specific characteristics that allow them to propose interventions to reduce snake bite.

I have a few comments, mostly minor

Major

1. It is easy to make recommendations, much more difficult to implement things. The authors have identified several interventions, none of which are particularly surprising or novel. I would like to know what overall number (%) of bites would be prevented by implementing their 5 recommendations

More importantly I would like to know how they intend to implement their recommendations? With whom, over how long? Does it needs funding. I would really like to know that they have this in process already; otherwise it risks becoming another epi paper that does not result in change or patient benefit. A Text box or figure in the Discussion laying out plans and time-lines would markedly increase the importance of the paper, especially when it is done and the effects are measured

Authors response:

We thank the reviewer for insightful comments on the outcome of this research and we accept the comment.

We have included the overall effect of applying all the recommended preventive measures in Table 2.

Additionally, we have included a Figure (Fig. 6) mentioning the way forward for community engagement of these preventive measures. At the initial stage, the researchers are conducting community-based surveys among high-risk populations for snakebite to gain insights into their snakebite practices and knowledge. This ongoing research by the researcher in this paper is being conducted among the community in the Anuradhapura district, Sri Lanka. Based on the results, the researchers intend to conduct socio-behavioural and feasibility studies among the key stakeholders (risk populations, agricultural officers, administrative officers) to identify the avenues for facilitating effective snakebite prevention in the region.

Fig. 6

| **1**  ━━━ | **Phase 1: Community Assessment** **ONGOING**  Moving beyond descriptive epidemiology, community-based cross-sectional surveys are being conducted among high-risk populations in Anuradhapura district to establish baseline data on preventive practices and behavioral barriers.  **Objective:** To quantify current adoption rates of evidence-based preventive measures and identify socio-behavioral barriers to implementation.  **Example focus:** *Protective footwear utilization during agricultural activities and determinants of non-compliance despite known D. russelii exposure risk.*  *Data collection in progress (2025-2026)* |
| --- | --- |
| **2**  ━━━ | **Phase 2: Analysis of socio-behavioural factors and feasibility for stakeholders** **PLANNED**  Multi-stakeholder feasibility studies will assess implementation pathways within existing community infrastructure and government programs.  **Objective:** To map sustainable integration opportunities and understand context-specific determinants of preventive behavior adoption across agricultural and domestic settings.  **Key stakeholders:**   - High-risk populations (farmers, rural residents) - Agricultural officers at ground level (for safety message integration) - District administrative officers (for policy and resource mobilization) - Educational institutions (for community outreach platforms)   *Projected timeline: 12–24 months post Phase 1* |
| **3**  ━━━ | **Phase 3: Intervention co-design and pilot testing** **PLANNED**  Evidence-informed interventions will be co-designed with communities and piloted to assess acceptability, feasibility, and preliminary effectiveness.  **Objective:** To develop and test culturally appropriate, context-specific prevention strategies informed by Phases 1–2 findings.  **Intervention examples:**   - Climate-appropriate protective footwear tailored for dry zone agricultural work - Targeted educational campaigns aligned with identified seasonal peaks (October–January, April–May) and high-risk activities - Developing long-handled tools and improved lighting in domestic gardens   *Projected timeline: 24–36 months post Phase 1* |
| **4**  ━━━ | **Phase 4: Policy translation and integration** **PLANNED**  Synthesized evidence from Phases 1–3 will be translated into actionable prevention frameworks for policy integration at the district and national levels.  **Objective:** To establish an evidence-based, culturally validated snakebite prevention framework for the Sri Lankan dry zone with clear implementation pathways.  *Projected timeline: 36–48 months post Phase 1*  **Expected outcome: Dissemination to the Ministry of Health, the Department of Agriculture, and provincial health authorities to incorporate findings into national snakebite prevention guidelines and agricultural safety protocols**. |

2. the abstract needs mildly improving (with implications for main text as well please)

a) “species-authenticated” needs defining here

Author's response:

We have defined the ‘species-authenticated’ in the abstract.

b) because of the high mortality with B caeruleus, pls provide the number of cases that occurred when sleeping (to accompany the ‘walking’ data – low numbers but will save lives)

Author’s response:

We have updated the Abstract to explicitly report the high proportion of *B. caeruleus* bites that occurred during sleep to emphasize this risk factor.

c) “and bites peaked from September to February” – can you indicate why here (was different for R viper I think)

Author’s response:

Overall, snakebite numbers are higher during this period. However, Russell’s viper bite patterns differ, as it is mainly a farming-related occupational hazard. In light of the reviewer's comment, we have edited the sentence to make it clearer.

“Increased numbers of bites were reported from September to February”.

d) “and hand bites occurred while cleaning” – cleaning what? Themselves (washing?) or brushing ground or picking up rubbish? Wld be good to get a better understanding of what action is meant by ‘cleaning’

Author’s response:

We have included this in the revised version of abstract

e) what total number of bites would be prevented by the 5 interventions

Author’s response:

Theoretical preventable proportion of snakebite by applying all five preventive measures include,

88.6% of all snakebites

93.4% of *H. hypnale* bites

92.7% of *D. russelii* bites

95.0% of *B. caeruleus* bites

77.4% of *N. naja* bites

81.5% of non and mildly venomous snakebites

We have modified the table 2, to include these findings in the manuscript.

f) the discussion says that thing shave changed since 1980s/90s. Would be nice to include this in the conclusions as it is important to understand change in epidemiology (authors argue reduced B caeruleus is due to change in sleeping habits)

Aurthor’s response:

We have included this information in the revised version of the abstract.

“Recent epidemiological patterns show a reduction of B. caeruleus and N. naja bite proportions with an increase in *H. hypnale* bite proportions.”

g) conclusion starts with: “We found simple measures could prevent many snakebites.” Not really - study identified epidemiology that indicates possible avenues for prevention. This study did not actually ‘find’ simple measures to prevent outdoor bites

Author’s response:

We thank the reviewer for pointing out this. We have changed the wording of the sentence according to the reviewer comment.

h) collecting firewood seems to be dangerous activity. However nmot sure how long -handed tools could help prevent these bites

Author’s response:

Two percent of all snakebite victims (and 7.7% of *H. hypnale* bite victims) experienced snakebites during firewood collection. Therefore, we agree with the reviewer’s statement, particularly regarding *H. hypnale* bites.

In this region, usually adults are involved in firewood collection from various places such as home garden, forests. The collected firewood is stacked to be used whenever necessary. We agree with the reviewer that using long-handled tools for lifting firewood is impractical. However, the intended preventive measure is to use a tool (such as a stick) to disturb or probe leaf litter and wood piles before reaching in with the hands. Since *H. hypnale* is highly camouflaged and often found in leaf litter, creating distance during this initial check can prevent bites to the fingers and hands.

Additionally, we plan to conduct a socio-behavioural study to explore practical ways to integrate such safety checks into the daily routine of firewood collection.

3.
“The remainder were bites due to unidentified snakes, 2495 (53·1%).”

How many of these patients were envenomed by main 4 but did not have either snake brought in or positive ELISA? Pls give numbers for this group by species and by non-envenomed or mildly-envenomed

Authors’ response

It is difficult to say how many of the unidentified snakebites were by the main 4; however, we have now updated the S1 table with the number of patients who had systemic/local envenoming vs dry bites, for both identified and unidentified snakebites.

4.
Figure 1 indicates that 116 were bitten while sleeping in the house. This differs from 96 autheticated bites and the 296 in all species in table 1. Please clarify

Author’s response:

In Table 1, the total number of snakebites that occurred was 296. Of those, 96 were authenticated bites. These 296 bites include the bites that occurred in all locations, such as indoors and farmlands. (Please note that local farmers frequently stay/sleep at the farmlands to protect their harvest from animals and thieves. These are usually open spaces with a shelter, as shown in Figure 5C. In Figure 1, our objective was to identify the main characteristics of snakebite based on bite location. In the Figure 1, 116 bites include only the hand and feet bites occurred indoors from 6pm to 6 am.

Minor

1.
“75 (74·3%) occurred at night (7 pm-6 am), and 47 (46·5%) while slept.”

60% bites occurred while sleeping, it is just that 47% occurred during night time hours. The tight link between ‘night’ and ‘slept’ is not clear. Could separate and show that 60% occurred while sleeping, which is the main point. Were these extra bites during mid-day, when I did not think that krait were about?

Of note the author summary makes this clear “Most Indian krait bites occurred late at night while victims were sleeping.”

Author’s response:

Since this sentence appears to cause confusion, we have modified it in the main text and the abstract.

“Of the 101 B. caeruleus bites, 75 (74·3%) occurred at night (7 pm to 6 am) (Fig 4). Sixty (60.6%) occurred while the patient slept.”

As for the clarification, out of these 60 bites, 47 occurred from 7 pm to 6 am, and the other 13 occurred from 6 am to 7 pm. Of those 13 bites, the majority occurred in the morning hours (6 am to 8 am) and early evening (6 pm to 7 pm).

2.
“with 398 snakebites and 151 snake envenomings per 100,000 population annually”

Wld be good to define bite vs envenoming, especially for mild snake bite. Were these patients envenomed or just bitten with local effects like pain?

Author’s response:

This sentence is a direct reference to the primary findings of the 2016 national survey by Ediriweera et al. In that original research, the authors distinguished between total 'snakebites' and 'significant envenoming' using the following criteria:

- Snakebite (Incidence: 398 per 100,000): This is defined as a bite event from any snake, regardless of whether the victim was actually envenomed. This includes all reported incidents, which may encompass dry bites or bites from non-venomous species where only local pain occurred without further systemic or tissue damage.
- Envenoming Bite (Incidence: 151 per 100,000): The researchers specifically defined this as a snakebite that resulted in significant envenoming. For the purpose of the study's statistics, significant envenoming was only recorded if the patient exhibited at least one of
  - Local tissue necrosis at the site of the bite.
  - Neurotoxicity.
  - Bleeding manifestations.

Therefore, the 151 envenomings represent the portion of patients who experienced these significant clinical effects, while the 398 figure reflects the total burden of all bite events reported by the community, including those that were mild or non-envenomed.

3.
“The diagnosis of snakebite is confirmed by the presence of fang marks and/or systemic or local clinical signs CONSISTENT WITH ENVENOMING”

Author’s response:

We have modified the sentence according to the reviewer’s suggestion.

4.
The term dusk is used in the paper, sometimes to indicate 18.00 to 21.00. Dusk is around 18.00 to 19.00 in SL. 19.00 to 21.00 is dark, after nightfall. So not really dusk – people should be using light sources at that time, so there is some nuance here

Author’s response:

We sincerely thank the reviewer for this valuable insight regarding the local context of twilight hours in Sri Lanka. We agree that the term 'dusk' is imprecise for the latter part of this time window (19:00–21:00), which represents full darkness and reliance on artificial lighting.

To address this, we have renamed the 18:00–21:00 category from 'Dusk' to 'Dusk and early night' throughout the manuscript.

We have also added a clarifying sentence in methods section acknowledging that this period encompasses both the brief twilight transition and the onset of nightfall where light sources become essential.

5.
“More bites were reported from March to May and from September to November, which is consistent with regional agricultural seasons,”

And
“In our cohort, bites by each of the snakes occurred during the peak of the Northeast monsoon rainy season. Further,”

These seem to clash and perhaps differ from the abstract (see comment #2 above)

Author’s response:

We have modified the relevant sentences to make them clearer.

“Bite reports peaked during two distinct intervals: March–May and September–November, coinciding with regional agricultural seasons. Therefore, the optimal strategy is to conduct community awareness programs leading up to and throughout these high-risk periods.”

“In our cohort, the frequency of bites for each medically important snake species increased during the Northeast monsoon rainy season.”

6.
“Prevention of bites on the hand from H. hypnale is particularly important in reducing morbidity, because H. hypnale is known to cause disabling local effects [8,24].”

Please say how common - how big a problem is it? If it is only 1 in 100, hard to make argument. If 30%+ of hand bites cause long term effects, then clearly major. Worth just adding % here to support your argument it is important

Author’s response:

We have included the information in the revised manuscript.

“Prevention of bites on the hand from H. hypnale is particularly important in reducing morbidity, because H. hypnale is known to cause disabling local effects in 0.7 - 18% of cases.”

7.
Table 2 – please add in an ‘all 5 measures’ and show how much be prevented if all done

Author’s response:

We have modified the table 2, to include these findings in the manuscript.

8.
Very nice illustrative photos in Figure 5

Author’s response

We thank the reviewer for their kind words regarding the illustrative photos in Figure 5. We are glad that these images effectively convey the practical context of snakebite risks in the study area.

Reviewer #2

**Methods**

1.The manuscript lacks sufficient justification for the geographic and population coverage. It is unclear whether the cases included are representative of all rural snakebite victims or only those seeking formal healthcare. Address selection bias explicitly and discuss how it may affect estimates of incidence, severity, and snake species distribution.

Author’s response:

We appreciate the reviewer’s emphasis on clarifying the population coverage and potential selection biases. We acknowledge that, as a hospital-based cohort, our data strictly represent those seeking formal healthcare.

However, we would like to point out that this cohort is highly representative of the region's snakebite burden due to two key factors:

1. Health-seeking behavior: In the North Central Province of Sri Lanka, reliance on allopathic (formal) medicine for snakebite is extremely high, minimizing the number of victims who solely consult traditional healers or stay home.
2. Centralized Care**:** The Teaching Hospital Anuradhapura is the only tertiary care centre in the district. While some minor cases may be managed at primary or secondary peripheral units, established referral protocols ensure that virtually all potentially severe or complicated cases are transferred to this treatment centre.

We have included an additional sentence in the methods to address the reviewer’s comment regarding the study population and geographic coverage.

“As the primary referral centre for the region, the hospital receives transfers from smaller peripheral units as well as direct admissions. Consequently, the study population consists exclusively of individuals seeking formal allopathic healthcare and does not include victims who solely utilized traditional treatments or did not seek medical attention.”

Regarding the estimates of incidence, severity, and snake species distribution, we wish to clarify that the primary objective of this study is not to calculate population-level incidence rates or analyze clinical severity outcomes. Instead, our goal is to identify epidemiological patterns (time of day, seasonal trends, and risk activities) to design effective community awareness and prevention strategies.

We acknowledge that as a tertiary care cohort, our data is subject to selection bias towards medically significant envenoming. Patients with dry bites or bites from non-venomous species may be under-represented as they are often managed at local peripheral units without transfer.

However, for the purpose of prevention planning, this bias is acceptable and perhaps even advantageous. The data accurately reflects the 'high-risk' bites—those requiring advanced medical care—which are the primary targets for mortality and morbidity reduction. Therefore, the risk behaviours (e.g., firewood collection, agricultural timing) identified in this cohort remain the most critical targets for community intervention.

We have added a statement in the ‘Discussion’ section explicitly noting that our species distribution reflects the hospital-presenting population rather than the total biological population of the region.

“This study did not capture all snakebites occurring in the district, as cases with mild or no envenoming may have been managed at secondary treatment centres without transfer. Consequently, the species distribution reported here mainly reflects the burden of medically significant snakebites requiring specialized management. While this study captures a substantial number of non-venomous or mildly venomous snakebite (n=314), these may still be under-represented. Additionally, snakebite deaths occurring before hospital admission were not included in this study. Therefore, the patterns described here may not completely reflect the overall snakebite epidemiology in the Anuradhapura district. However, we believe the majority of significant cases were included. Therefore, the epidemiological patterns described here represent the priority targets for preventing severe morbidity and mortality.”

2.The method of snake species identification appears to rely largely on patient reports or indirect evidence. This raises concerns about misclassification bias, especially in rural settings where snake recognition is unreliable. Clarify whether expert verification, photographs, or venom detection methods were used.

We thank the reviewer for highlighting the critical issue of species misclassification, especially in rural settings where lay identification can be unreliable.

We wish to clarify that species identification in this cohort was **not** based on patient verbal reports or indirect evidence. As described in the Methods section, species were rigorously authenticated using two reliable methods:

1. Herpetological Examination: Dead or live specimens brought by patients were examined by a herpetologist (Prof. Anjana Silva).
2. Venom Detection: In cases where the snake was not brought, species were identified via enzyme-linked immunosorbent assay (ELISA) for venom detection.

We have slightly revised the Methods section to explicitly state that patient self-reporting was not used as a primary method for species classification.

“Snake species are authenticated either by examination of snake specimens by a herpetologist (AS) or by venom detection in serum samples from patients using enzyme-linked immunosorbent assay (ELISA) [20–22]. Species identification was not based on victim or bystander testimony. Data were entered into a relational electronic database.”

**Results**

The reviewer has no comments on the results

**Conclusions**

The reviewer has no additional comments on the conclusion

**Summary and General Comments**

This manuscript addresses snakebite epidemiology in rural Sri Lanka, a topic of high relevance to neglected tropical diseases, occupational health, and rural public health. Given Sri Lanka’s well-documented burden of snakebite envenoming, the study has the potential to contribute meaningful evidence to guide prevention strategies and policy interventions. The dataset appears substantial, and the authors attempt to link epidemiological patterns with practical preventive implications, which aligns well with the journal’s translational focus.
However, while the study is valuable, several conceptual, methodological, and interpretative limitations reduce its current impact. In particular, the definition of novelty, clarity of study design, analytical depth, and strength of inference regarding preventive measures require significant improvement. The manuscript would benefit from a more rigorous epidemiological framework and a clearer separation between descriptive findings and policy recommendations.

1.The manuscript does not clearly define whether the study is prospective, retrospective, or cross-sectional. The authors should explicitly clarify:
λWhat hypotheses were tested (if any)?
λWhether the primary goal was descriptive epidemiology or evaluation of risk factors.
Reframe the Introduction to clearly articulate the study design, primary outcomes, and how these differ from prior snakebite studies in Sri Lanka.

Author’s response:

We thank the reviewer for this suggestion to improve clarity.

We confirm that this is a prospective cohort study (Anuradhapura Snakebite Cohort). We did not test formal pre-specified hypotheses. Instead, our primary goal was descriptive epidemiology to characterize the distinct circumstances of bites for each major snake species in a contemporary cohort. As requested, we have reframed the final paragraph of the Introduction to explicitly state:

1. The study design (prospective cohort).
2. The primary objective (descriptive epidemiology to identify prevention targets).
3. The distinction from prior work (providing updated data on species-specific patterns in the context of modern socio-economic changes, unlike older retrospective studies)."

We modified the introduction according to the reviewer comment.

“To address these gaps, we analysed data from the Anuradhapura Snakebite Cohort, a large prospective study in rural Sri Lanka. The primary objective of this descriptive study was to characterize patterns of snakebite exposure across different snake species and to identify potential preventive strategies targeting observed high-risk circumstances. Unlike previous retrospective or smaller studies, we focused on the species-specific characteristics of snakebites (location, bite site, activity, and time of day) to identify theoretical targets for prevention.”

2.The manuscript lacks sufficient justification for the geographic and population coverage. It is unclear whether the cases included are representative of all rural snakebite victims or only those seeking formal healthcare. Address selection bias explicitly and discuss how it may affect estimates of incidence, severity, and snake species distribution.

Author’s response

We have modified our manuscript in response to the reviewers' comments. The detailed response was included as the response for the reviewers’ comment 1 in the methods.

3.The method of snake species identification appears to rely largely on patient reports or indirect evidence. This raises concerns about misclassification bias, especially in rural settings where snake recognition is unreliable. Clarify whether expert verification, photographs, or venom detection methods were used.

Author’s response

We have modified our manuscript in response to the reviewers' comments. The detailed response was included as the response for the reviewers’ comment 2 in the methods.

4.Several key Sri Lankan and South Asian snakebite studies are cited, but comparison is largely superficial. Differences and similarities with prior national surveillance data are not sufficiently explored. Expand the Discussion to compare:
λIncidence patterns
λSpecies distribution
λTemporal trends
with previous Sri Lankan studies and WHO snakebite frameworks.

Author’s response:

We thank the reviewer for this valuable suggestion. We agree that placing our findings within the broader context of national surveillance and global strategies strengthens the manuscript.We have expanded the Discussion section to provide a more robust comparative analysis:

1. We have added text explicitly comparing our species distribution with an island-wide snakebite survey. We highlight that while *H. hypnale* is the leading cause nationally. Our cohort records a significantly higher proportion of *D. russelii* bites (33%) compared to the national average, reflecting the unique ecological risks of the dry zone agricultural hub.
2. We have synthesized historical data to discuss the "divergent trends" in the region: specifically, the marked decline in *B. caeruleus* bites (attributed to improved housing over the last two decades) versus the persistence of viper bites (attributed to continued agricultural exposure).
3. We have added a statement linking our findings to the World Health Organization’s strategy for snakebite prevention and control. We specifically note how our identification of local, species-specific risk patterns supports the WHO strategic pillar of "empowering communities" by moving beyond generic advice to targeted, culturally relevant interventions.

The changes to the manuscript as follows:

“This species distribution aligns with the findings of an island-wide snakebite survey, which identified *H. hypnale* as the commonest cause of snakebites [4]. However, our cohort records a higher proportion of *D. russelii* bites (33%) than the national average, reflecting the specific ecological conditions of the dry-zone agricultural areas.”

“This represents a notable temporal shift of the epidemiological profile in the region. While viper bites (linked to engaging in outdoor and farming activities) have persisted, krait bites (linked to housing conditions) have declined.”

“These findings directly support the World Health Organization’s strategy for snakebite prevention and control [3], specifically the strategic pillar of 'empowering communities.' By shifting focus from generic advice to species-specific targets (e.g., footwear for vipers vs. safe sleeping for kraits), these data facilitate the development of culturally relevant, community-based interventions.”

5.The manuscript would benefit from professional English editing. Several sentences are long, repetitive, or unclear.

Author’s response

We thank the reviewer for this observation.

We have conducted a comprehensive review of the entire manuscript to improve clarity and flow. Specifically, we have:

1. Simplified complex sentences: Long sentences in the Introduction and Discussion were split to improve readability.
2. Removed repetition: We ensured that findings reported in the Results were not redundantly restated in detail in the Discussion.
3. Improved clarity: Key sections, particularly the definitions of study categories and the implementation roadmap (Fig. 6), have been refined for precise expression.

We believe the language is now concise and meets the professional standards of the journal.

Reviewer #3: 

**Methods**

Objectives are SMART

Yes methodology is appropriate
Clearly outline the sample population

**Results**

Yes all appropriate

**Conclusions**

Limitations not clearly stated especially the number of deaths as a result of snakebites

Author’s response:

We thank the reviewer for pointing out this omission. We agree that it is crucial to explicitly state the limitations regarding mortality data, particularly deaths that occur in the community.

We have revised the 'Strengths and Limitations' section to clearly state that snakebite deaths occurring prior to hospital admission are not captured in this dataset. We have also clarified that our data reflects outcomes for patients reaching tertiary care, which may differ from overall district-level mortality.

“This study did not capture all snakebites occurring in the district, as cases with mild or no envenoming may have been managed at secondary treatment centres without transfer. Consequently, the species distribution reported here mainly reflects the burden of medically significant snakebites requiring specialized management. While this study captures a substantial number of non-venomous or mildly venomous snakebite (n=314), these may still be under-represented. Additionally, snakebite deaths occurring before hospital admission were not included in this study. Therefore, the patterns described here may not completely reflect the overall snakebite epidemiology in the Anuradhapura district. However, we believe the majority of significant cases were included. Therefore, the epidemiological patterns described here represent the priority targets for preventing severe morbidity and mortality.”

**Summary and General Comments**

none

Reviewer #4:

**Methods**

1. Line 116. Please consider showing the study site (the hospital) within Sri Lanka using a map.

Author’s response:

We thank the reviewer for this helpful suggestion. We agree that a geographical representation aids the interpretation of the study context for international readers.

We have created and included a new map as a supplementary figure (S1 Fig) that highlights the location of the Teaching Hospital Anuradhapura within the North Central Province of Sri Lanka. This figure clearly illustrates the study setting in relation to the island's geography.

2. Line 119-125. Please add clearer definitions for key variables: time of day (including what "dusk" means), season/month groupings, activity (whether one main activity was recorded or multiple responses were allowed), location categories (e.g., garden/farmland/paddy/chena-please define "chena"), and bite site grouping (hand/foot/other).

Author’s response:

We thank the reviewer for this constructive feedback. We agree that explicit definitions are essential for readers—particularly those unfamiliar with the region—to fully understand the study context and categorization.

We have revised the 'Materials and Methods' section (under Data retrieval and variable definitions) to provide clear definitions for all the variables requested.

“To ensure clarity within the regional context, variables were explicitly defined and categorized. The activity the victim was performing at the time of the bite was recorded as a single primary activity, and multiple responses were not permitted. Locations were classified into distinct groups, including home gardens, farmlands [paddy fields (wet farming lands), Chena (traditional slash-and-burn shifting cultivation) and other farmlands (banana fields, coconut estates, etc.)], indoors, roadside, and other locations. For temporal analysis, the period from 18:00 to 21:00 was defined as 'dusk and early night', while seasonal trends were analyzed based on calendar months. Anatomical bite sites were grouped into the foot, hand, leg and other sites.”

3. Line 123-125. Species-specific analyses appear to rely on the specimen/ELISA-authenticated subset. However, approximately 53% of cases did not have species identification reported. Please explain why many cases were not authenticated (e.g., no specimen, unidentifiable specimen, no sample/ELISA) and consider a brief comparison of authenticated vs non-authenticated cases (age/sex, indoor/outdoor, time of day, bite site) to assess potential selection bias.

Author’s response:

We appreciate the reviewer’s insightful comment regarding the authentication of snake species and the potential for selection bias.

In the majority of the unidentified snakebite instances, the snake was not captured/killed for identification. To assess potential selection bias, we have performed a comparison between the authenticated and non-authenticated (unidentified) snakebite cases across several key demographic and environmental variables, including age, sex, location (indoor/outdoor), time of day, and bite site. As per the suggestion, we have included these comparative data in the Supplementary S1 Table. We chose to place this detailed comparison in the supplementary material rather than in Table 1 to maintain the readability and focus of the primary manuscript, as adding several additional columns for unidentified bites would significantly increase the complexity of the main table.

Findings: Our analysis of the S1 Table demonstrates that there are no significant differences between authenticated and non-authenticated cases across these variables, suggesting that the subset of cases with identified species is representative of the overall bite burden and that significant selection bias is unlikely.

4. Line 118-119. Recruitment spans Aug 2013-Oct 2014 and then resumed from May 2017; please state the end date alongside these periods (you later mention Jan 2023, but it is clearer to present the full timeframe in one place). Please also note how this pause may affect interpretation of seasonal patterns and trend comparisons over time, and briefly describe how you addressed this in the analysis.

We thank the reviewer for requesting clarification on the timeframe and for this important observation regarding potential bias in the seasonality analysis caused by the data gap.

We have updated the Methods section to explicitly list the recruitment periods: Phase 1 (Aug 2013 – Oct 2014) and Phase 2 (May 2017 – ongoing), with the data retrieval cutoff for this study set at 31st January 2023. We have added a specific statement in the 'Data Analysis' section describing this inclusion criterion of the study.

“Recruitment spanned two phases: 1st August 2013 to 31st October 2014, and continuing from 1st May 2017 onwards. Although the database is ongoing, data for this overall analysis were retrieved up to 31st January 2023.”

We wish to clarify that in our originally submitted analysis, we had already accounted for this issue. To ensure the accuracy of seasonal trends, we specifically restricted the seasonality analysis to the continuous five-year period from 1st January 2018 to 31st December 2022. This approach ensured that all seasonal patterns reported in the manuscript were derived from an uninterrupted dataset, effectively eliminating any bias from the recruitment hiatus.

We acknowledge that this distinction was not explicitly stated in the previous Methods section. We have now revised the 'Data Analysis' subsection to clearly specify that seasonal analysis was restricted to this continuous 2018–2022 timeframe, while the overall demographic analysis utilized the full dataset up to January 2023."

5. Line 134-136. You report "valid percentages" excluding missing data; please also report the amount of missing data and denominators (n/N) for key variables later in the result ( and tables, figures).

Author’s Response:

We thank the reviewer for this suggestion to enhance the transparency and clarity of our data reporting. We have revised the results section and all corresponding tables and figures to explicitly include the amount of missing data for each variable. As requested, we have added a new column specifically for missing data to each relevant table. We continue to provide "valid percentages" (percentages calculated after excluding missing values) to reflect accurate clinical or demographic trends.

**Results**

6. Table 1.
a. Sample sizes (N) differ across variables; please explain this clearly in the caption or footnotes, and report denominators consistently (n/N) with missingness highlighted.

Author’s response:

We appreciate the reviewer’s attention to detail regarding data completeness.

We have revised the table accordingly.

1. Explicit Denominators: We have updated Table 1 to include specific sample sizes (n=...) in the sub-header for each variable (e.g., Gender, Occupation). This clarifies the exact denominator used for percentage calculations in every section.
2. Missing Data Highlighted: As requested, we have added a dedicated "Missing data" row at the bottom of each section to explicitly quantify gaps in the medical records.
3. Footnote: We have revised the table footnote to explain that sample sizes vary across variables due to incomplete documentation, and that percentages are calculated based on the available data (n) rather than the total cohort (N).

b. Location categories appear overly granular and some (e.g., "chena") are not defined; consider collapsing categories and defining all terms in Methods.

We thank the reviewer for this suggestion.

As requested, we have added a clear definition of **'**Chena' (traditional slash-and-burn shifting cultivation) to the 'Data retrieval and variable definitions' subsection in the Methods to ensure clarity for international readers.

Regarding the suggestion to collapse location categories, we carefully considered this but respectfully believe that retaining the distinct categories (Home Garden, Paddy Field, Chena) is essential for the epidemiological accuracy of this study. In the dry zone of Sri Lanka, a 'Paddy Field' (wetland agriculture) and a 'Chena' (dryland slash-and-burn cultivation) represent vastly different ecological niches. Our data shows distinct variations in snake species distribution across these locations (e.g., *D. russelii* is predominantly associated with paddy fields, while *H. hypnale* is frequently found in home gardens).

Collapsing these into a single 'Agriculture' category would obscure these specific habitat-species associations, which are critical for developing targeted prevention strategies (e.g., advising footwear specifically for paddy farmers vs. home gardeners).

c. Activity categories may overlap (e.g., walking during cleaning/gardening); please clarify in the Methods whether one main activity was recorded or multiple responses were allowed, and how percentages were calculated.

Author’s response:

We appreciate the reviewer’s comment regarding the potential overlap of activity categories (e.g., walking vs. gardening).

We have clarified in the 'Materials and Methods' section that the activity variable captured the single primary activity the victim was engaged in at the time of the bite. In cases of overlap (e.g., walking while farming), the specific occupational or purposeful task (Farming) took precedence over the general action (Walking). Multiple responses were not permitted, which prevents statistical overlap.

Regarding the calculation of percentages, we used valid percentages. This means the denominator for each activity category excludes missing data or 'unknown' responses, ensuring that the reported proportions accurately reflect the known distribution of risk activities.

Revised text in manuscript:

'The activity performed by the victim at the time of the bite was recorded as a single primary activity, and multiple responses were not permitted... When indicating the percentage of data, we present the valid percentages of the variables, disregarding missing data on relevant variables.'"

d. Where differences between species or categories are discussed, please either support them with simple statistical comparisons (e.g., Fisher's exact or chi-square tests) or rephrase as descriptive patterns (e.g., "in this cohort, XX% occurred" / "most frequently reported" / "the majority occurred").

Author’s response:

We appreciate the reviewer’s suggestion to distinguish between statistical significance and descriptive observations.

Given that the primary objective of this study is to describe the epidemiological context and identify practical preventive avenues (rather than testing specific hypothesis-driven group differences), we have opted for the reviewer’s second suggestion: presenting these findings as descriptive patterns.

We have reviewed the Results and Discussion sections to ensure the language reflects this descriptive approach. We have avoided terms implying statistical inference (e.g., 'significantly different') and instead used language focused on observed proportions and frequencies (e.g., 'The majority of bites occurred...', 'X was the most frequently reported location...'). This accurately conveys the magnitude of the risk without overstepping into unsupported statistical claims.

7. Table 2 (in discussion).
a. It currently implies intervention effectiveness or "% preventable" (e.g., "60% potentially prevented"). As this is a descriptive study, the manuscript should avoid predicting prevention impact (e.g., "X% preventable") and instead describe prevention opportunities suggested by the observed patterns. I suggest reframing this table as "prevention opportunities/targets suggested by observed bite circumstances" and removing quantitative "% prevented" claims.

Author’s response:

We thank the reviewer for this thoughtful comment regarding the distinction between descriptive data and intervention effectiveness.

While we agree that this is a descriptive study, identifying the 'preventable fraction' (the proportion of cases occurring under specific, modifiable circumstances) is a standard and critical output for public health epidemiology. These percentages do not predict the efficacy of a future trial, but rather quantify the burden of risk that could theoretically be addressed by specific interventions (e.g., 83% of *D. russelii* bites occur on the feet/ankles).

We would also like to note that other reviewers specifically found these quantitative estimates to be a strength of the manuscript, with one reviewer requesting even further aggregation of these preventable percentages. Removing them would reduce the practical utility of the study for local health policy planners.

To address your concern about clarity, we have slightly modified the column header in Table 2 to 'Theoretical Preventable Fraction' and added a footnote clarifying that these figures represent the proportion of bites occurring in circumstances modifiable by the intervention. This balances the need for caution (your point) with the need for quantitative targets (the other reviewers' points).

8. Figures 1 and 2. They are difficult to interpret because denominators appear to change and branches may not sum to 100%. The figures are overly complex, with multiple layered categories, which makes them difficult to interpret. Consider simplifying to stacked bar charts/heatmaps (e.g., species by location/activity/time).

Author’s response:

We thank the reviewer for their feedback on the interpretability of Figures 1 and 2.

We chose flow charts over stacked bar charts because our specific aim was to visualize the pathway of risk [the connection between Activity and Anatomical Site related to bite location (Figure 1), and snake species (Figure 4)] for the same group of patients. A stacked bar chart would decouple these variables, losing the ability to see how specific activities lead to specific bite locations.

We wish to clarify that the branches do not sum to 100% by design. To ensure the figures remained readable and focused on high-priority targets for prevention, we intentionally omitted minor/low-frequency categories from the visualization. Furthermore, the percentages presented are calculated based on the starting N (total group) rather than a changing denominator at each branch. This ensures that a percentage at the end of a branch (e.g., '20% bitten on foot') is directly comparable to the total population, minimizing confusion.

As three other reviewers found these figures to be clear and suitable for the manuscript, we have elected to retain the flow chart format. However, to address your concern about interpretability, we have added a clarifying note to the figure legends stating: 'Percentages are calculated based on the starting N; branches may not sum to 100% as minor categories were omitted for visual clarity*.’*

9. Figure 3. The recruitment pause should be considered in this analysis. Consider presenting counts by year and month, or otherwise clarify how months/years with incomplete coverage were handled (with explanation).

Author’s response:

We thank the reviewer for highlighting the potential bias in Figure 3 due to the recruitment pause. As noted in our response to the previous comment regarding the study timeframe, we have specifically addressed this issue in the analysis design. Figure 3 (seasonal trends) was generated using data strictly restricted to the continuous five-year period from 1st January 2018 to 31st December 2022.

By restricting the analysis to this continuous block, we ensured that every calendar month is equally represented (exactly five of each month: 5 Januaries, 5 Februaries, etc.). This eliminates the need to normalize for incomplete years or the recruitment pause, as the data used for this figure contains no gaps.

We have added a statement in the 'Data Analysis' section confirming that seasonal patterns were derived exclusively from this continuous timeframe."

**Conclusions**


10. Discussion (overall). The Discussion contains several statements that read as causal or risk-factor conclusions without supporting analysis. Please soften wording to reflect descriptive patterns and clearly state that prevention implications are potential targets rather than quantified effects.

We accept this constructive feedback. We agree that, as a descriptive study without a control group, our language should focus on observed epidemiological patterns rather than causal risk factors or quantified intervention effects.

We have carefully reviewed the Discussion section and 'softened' the wording as requested.

We have rephrased statements implying direct causality or 'risk' to instead describe coincidence or observed associations (e.g., changing 'risk factors' to 'observed circumstances'). We have removed predictive language (e.g., 'X% would be prevented') and replaced it with descriptive language identifying the proportion of bites occurring in modifiable circumstances (e.g., 'X% of bites occurred on the feet, suggesting footwear as a relevant target'). As noted in our response to Comment 7, we have renamed the 'Likely Impact' column to 'Theoretical Preventable Fraction' to emphasize that these are theoretical targets based on descriptive data.

11. Limitations (Line 314-320). On the current limitation statement, please also add some issues such as: hospital-based selection, species authentication in only a subset, interrupted recruitment affecting seasonality, and potential recall or category misclassification for activity, time, and location.

Author’s response:

We thank the reviewer for pointing out areas for improvement. We have modified the ‘Discussion’ section addressing the reviewer’s concerns.

“This study did not capture all snakebites occurring in the district, as cases with mild or no envenoming may have been managed at secondary treatment centres without transfer. Consequently, the species distribution reported here mainly reflects the burden of medically significant snakebites requiring specialized management. While this study captures a substantial number of non-venomous or mildly venomous snakebite (n=314), these may still be under-represented. Additionally, snakebite deaths occurring before hospital admission were not included in this study. Therefore, the patterns described here may not completely reflect the overall snakebite epidemiology in the Anuradhapura district. However, we believe the majority of significant cases were included. Therefore, the epidemiological patterns described here represent the priority targets for preventing severe morbidity and mortality.”

12. The aim (Line 101-105) is appropriate for the sample size, but to match this aim, the manuscript should clearly present findings as descriptive patterns unless formal statistical comparisons are added.

Author’s response

We thank the reviewer for confirming the appropriateness of our study aim. To ensure the manuscript aligns strictly with this descriptive aim, we have carefully reviewed the Results and Discussion sections.

As noted in our responses to previous comments, we have:

1. Removed inferential language: We avoided terms like 'significantly' or 'risk factor' where formal statistical comparisons were not performed.
2. Focused on patterns**:** We rephrased findings to highlight observed frequencies and proportions (e.g., 'predominant location,' 'observed percentage') rather than implying causal conclusions.

We believe the revised text now accurately reflects the descriptive nature of the study as outlined in the aim.

**Summary and General Comments**
This manuscript uses a large hospital-based cohort to describe snakebite circumstances and, where available, specimen/venom-based identification of the biting species. The descriptive dataset has clear potential value for informing prevention messages in the region. However, the current Methods and presentation lack key variable definitions and denominators, and parts of the Results/Discussion move beyond descriptive reporting to imply associations and prevention impact without supporting analyses. Clarifying methods, improving presentation, and tempering over-interpretation would substantially strengthen the manuscript.
